# Carcinogenic and neurotoxic risks of dietary acrylamide consumed through cereals among the Lebanese population

**DOI:** 10.1186/s13065-020-00705-2

**Published:** 2020-08-19

**Authors:** Areej Merhi, Ghada El-Zakhem Naous, Ralph Daher, Martine Abboud, Mohamad Mroueh, Robin I. Taleb

**Affiliations:** 1 School of Arts and Sciences, Department of Natural Sciences, Lebanese American University, Byblos, Beirut, Lebanon; 2 Department of Chemistry, University of Oxford, Oxford, UK; 3 School of Pharmacy, Department of Pharmaceutical Sciences, Lebanese American University, Byblos, Lebanon

**Keywords:** Cereals, Acrylamide, Carcinogenic, Neurotoxic, LC–MS/MS, WHO

## Abstract

The present study aims to determine the carcinogenic and neurotoxic risks associated with acrylamide intake from cereal products. Analysis on a UPLC–MS/MS spectrometer revealed that oat-based and mixed cereals contain the highest amount of acrylamide among cereal products with levels as high as 271 and 348 μg/kg, respectively. Children were shown to exhibit both carcinogenic and neurotoxic risks regardless of the type of cereal product consumed. For adults above 50 years of age, only consumers of oat-based cereal products seem to exhibit carcinogenic and neurotoxic risks. To avoid a carcinogenic and neurotoxic risk among the Lebanese population, we propose that food processors set the maximum tolerable concentration for acrylamide in cereal products at 94.8 μg/kg product, a value which is threefolds lower than the average acrylamide levels found in this study. Alternatively, and unreasonably, the average Lebanese population and children among the Lebanese population may choose to cut down on cereal consumption by 1.7- and 7.2-folds respectively, should they want to avoid a health hazard as a result of acrylamide intake. The industry should also respond by optimizing the production process in a way to reduce acrylamide levels in cereals.

## Introduction

A diet that is based on highly processed food may indeed provide nutrients, yet it will also provide many other compounds that are formed during the processing and storage of such foods [1]. Accordingly, standard industrial procedures are usually followed to minimize the formation of contaminants as byproducts. Contaminants maybe introduced from an external source or as a result of microbial, fungal, or animal activity [2]. Processing contaminants are undesirable either because they have an adverse effect on product quality or because they are potentially harmful [2]. Since the mid twentieth century, technological development in the food preparation process has contributed significantly in the reduction of the levels of processing contaminants [1].

Acrylamide, a processing contaminant formed during the cooking or high temperature processing of mainly plant derived foods and present in popular food, is now on the most pressing problems facing the food industry [3, 4]. Acrylamide is a difunctional monomer [5] and is used as a chemical for the production of polyacrylamide.

Acrylamide is formed at temperatures above 120 °C in thermal food treatment such as frying, baking and roasting and to a lesser extent boiling [6]. The toxic chemical is principally formed via the Maillard reaction and mainly in foods containing free asparagine and reducing sugars [7–9]. Acrylamide is found in many types of food where the processing involves thermal treatment such as coffee, biscuits, crackers, chips, cereals, rice cakes, and bread and its derivatives.

Since the detection of acrylamide in food in 2002 [10], acrylamide levels in various types of food products were monitored in various countries and the findings prompted a public health concern [11]. As a result, several research-based stakeholder meetings, workshops, and forums were held in order to shed light on the toxicity of acrylamide and the importance of reducing acrylamide levels in various food products [11]. Additionally, the FDE (FoodDrinkEurope) developed the ‘AA toolbox’ which is a regulatory updated guide for industry with possible intervention steps for reducing acrylamide levels in food [12]. In addition, the Belgian Federal Agency for the Safety of Food has monitored acrylamide levels in food and enforced industrial procedural regulations in order to reduce acrylamide levels in food products [7]. Sansano et al. [13] investigated acrylamide levels in fries and concluded that air-frying reduces acrylamide levels relative to deep oil-frying.

The European commission has established mitigation measures and benchmark levels for the reduction of acrylamide in food products [14]. A recent study carried out in our laboratories, revealed a high acrylamide content in potato/corn-based chips as well as in caffeinated beverages sold in the Lebanese market [15, 16]. In addition to the high consumption of chips and caffeinated beverages among the Lebanese population, there also seems to be a high dietary intake of cereals.

The present study aims to determine the carcinogenic and neurotoxic risks associated with the daily consumption of acrylamide from local and imported cereal brands as well as suggest the need for potential industrial regulations that would help minimize acrylamide levels in food products.

## Materials and methods

### Acrylamide analysis

Cereal products, both local and imported, were randomly collected from several locations in Lebanon. Samples of different brands were collected and analysed as duplicates with different production dates. In total, 49 samples were collected and classified as follows: corn, rice, wheat, oat or mixed based cereals.

#### Chemicals and reagents

All reagents and chemicals utilized in the analyses of acrylamide were of analytical grade. Acrylamide (≥ 99%), *n*-hexane (95%), dichloromethane (LC–MS grade), propan-1-ol (99%) and acetonitrile (LC–MS grade) were purchased from Merck, Germany. Distilled water (type II +) was prepared using a Millipore MilliQ system.

#### UPLC-MS/MS analysis

Acrylamide was extracted from cereal products according to previously reported methods [17]. Cereal samples (0.5 g each) were mixed with water (5 mL) and heated at 70 °C for 30 min. Propanol (10 mL) was added and the mixture centrifuged at 2200 rpm and 20 °C for 10 min. The aqueous supernatant was concentrated under reduced pressure before acetonitrile (2 mL) and hexane (5 mL) were added and the mixture sonicated for 3 min. The organic acetonitrile layer was re-extracted with hexane (5 mL), filtered using PTFE Millipore 0.25 µm filter and analyzed on a UPLC-MS/MS TSQ Endura Triple Quadruple Mass Spectrometer (Thermo Fisher Scientific, Waltham, MA) using a C18-Hypersil Gold reverse phase column. Analysis sample (10 µL) was injected and chromatographic separation was carried out in 2.5 min using an isocratic mobile phase of 5% water in methanol at a constant flow rate of 0.25 mL/min. MS/MS analysis was carried out in APCI mode under the following parameters: Positive Ion Discharge Current, 1.5 µA; sheath gas, 25; auxiliary gas, 5; sweep gas, 0; ion transfer tube temperature, 325 °C; and vaporizer temperature, 350 °C. Analysis of acrylamide was carried out using an SRM experiment at a retention time of 1.4 min and CID Gas of 1.5 mTorr whereby the transition from 72.3 → 55.3 (CE 10 V) was monitored. Quantification of acrylamide was carried out against a 4-point calibration curve (*R*^*2*^ 0.9971) constructed using standard acrylamide solutions of 100, 50, 25 and 10 ppb (µg/L). The limit of quantification (LOQ) was found to be 5.0 ppb while the limit of detection (LOD) was found to be 0.1–0.5 ppb. Acrylamide recovery was 92% which is higher than the reported average of 70-80% [17].

### Community survey

To determine the national consumption of cereals, an online survey (via the Lebanese American University’s Social media platforms) targeting the Lebanese population was conducted between December 2017 and April 2018 in which 845 anonymous responses were recorded. The target population pertains to Lebanese residents from the ages of 3–75 and residing across the entire geographical area of Lebanon. Subjects were interviewed about age, gender, body weight, type of cereals, reason of choice, in addition to daily recall consumption. The national survey was pre-approved by the International Review Board at the Lebanese American University (IRB #: LAU.SAS.RT1.1/Aug/2017). Consent from all participants was acquired through the survey as approved by the IRB committee.

### Dietary exposure assessment

The daily intake of acrylamide was calculated using our previously published procedure [16] by taking into consideration the consumers body weight. The assessment of neurotoxic and carcinogenic risks was carried out using our previously published procedure in which we determined the margin of exposure (MOE), no-observed-adverse-effect level (NOAEL) and the benchmark dose for a 10% response (BMDL_10_) [16, 18, 19]. The NOAEL value used for the assessment of neurotoxic risks was that set by the FAO (Food and Agriculture Organisation) and WHO (World Health Organisation) Expert Committee on Food Additives (JECFA) and stands at 0.2 mg/kg-bw/day for morphological changes in rat nerves [18]. For the assessment of carcinogenic risks, the BMDL_10_ values used in this study were 0.31 mg/kg-bw/day (mammary tumors in rats) and 0.18 mg/kg-bw/day (Harderian gland tumors in mice) as reported by JECFA [18].

### Statistical analysis

Statistical analyses were performed using Microsoft Excel 2016 software (Excel, Redmond, WA, USA). The results were analyzed for statistical significance using t-tests. Values of the different tested parameters within each group are presented as mean ± SEM and differences between groups were considered statistically significant if p-value < 0.05.

## Results and discussion

### Acrylamide analysis

Duplicate samples of cereal products with different production dates were extracted and analyzed for their acrylamide content. LC–MS analysis revealed that the average level of acrylamide was higher than the LOQ in all the tested samples. Results (Table 1) are reported according to the type of cereals (corn, wheat, rice, oat and mixed) and considering a recovery rate of 92%. The average value of acrylamide content was considered as variations were observed. Such variations could be attributed to variations in cereal brands, variance in food precursors, and most notably the lack of a unified industrial that should be followed by quality control prior to delivering a product to the market. Notably, Table 1 shows that corn and rice based cereal products contain the lowest mean acrylamide levels, a result that could be correlated to the fact the corn and rice grains are low in asparagine [14].

**Table 1:** Statistical analysis of acrylamide content (μg/kg) from different cereal types as obtained from duplicate analysis

Group	Mean ± SEM (95%)	Median	Maximum	Minimum	n
Corn based	219.91 ± 67.41	212.74	373.25	140.93	10
Wheat based	255.94 ± 132.78	246.86	445.16	81.82	10
Oat based	271.53 ± 111.05	266.57	479.03	127.80	11
Rice based	237.27 ± 123.16	261.31	393.05	93.38	8
Mixed	348.49 ± 146.90	415.16	485.87	117.78	10
Total	267.93 ± 122.50	246.71	485.87	81.82	49

Corn based cereal samples had acrylamide content that ranged from 140.93 to 373.25 μg/kg with an average value of 219.91 μg/kg and a median of 212.74 μg/kg (Table 1). The average concentration of acrylamide at the 95% confidence level was found to range between 171.73 μg/kg and 269.09 μg/kg (t-statistic). In 2006, Spain has reported an average for corn based cereals to be 207 μg/kg which is comparable to our study [20]; however more recently Mesias have shown that the mean acrylamide content for corn based cereals in Spain was significantly lower at 64 μg/kg [21]. Among the Taiwanese population, Cheng reported the mean acrylamide intake from corn based cereal at 271 μg/kg, a result which is comparable to that shown in this study [22].

Wheat based cereal samples had acrylamide content that ranged from 81.82 to 445.16 μg/kg with an average value of 255.94 μg/kg and a median of 246.86 μg/kg (Table 1). The average concentration of acrylamide at the 95% confidence level was found to range between 161.05 μg/kg and 350.83 μg/kg (t-statistic). Our values are lower than those reported in Spain where an average of 382 μg/kg was detected [20], while in Taiwan the average was found to be 305 μg/kg [22].

Oat based cereal samples had acrylamide content that ranged from 127.80 to 479.03 μg/kg with an average value of 271.53 μg/kg and a median of 266.57 μg/kg (Table 1). The average concentration of acrylamide at the 95% confidence level was found to range between 196.86 and 346.2 μg/kg (t-statistic) which is 2.8- to 4.9-folds higher than that reported among the Spanish community [21].

Rice based cereal samples had acrylamide content that ranged from 93.38 to 393.05 μg/kg with an average value of 237.27 μg/kg and a median of 261.31 μg/kg (Table 1). The average concentration of acrylamide at the 95% confidence level was found to range between 134.51 and 340.03 μg/kg (t-statistic). The average is 1.8- to 4.6-folds higher than that reported among the Spanish community (74 μg/kg) [21] and 5.4- to 13.6-folds higher than the average reported among the Taiwanese community which stands at 25 μg/kg [22].

Mixed grain cereal samples had the highest acrylamide content that ranged from 117.78 to 485.87 μg/kg with an average value of 348.49 μg/kg and a median of 415.16 μg/kg (Table 1). The average concentration of acrylamide at the 95% confidence level was found to range between 243.5 and 453.48 μg/kg (t-statistic); a result compared to those reported in Spain where average acrylamide levels were found to stand at 307 μg/kg [20].

In 2014, Colombia reported the average of acrylamide in cereals to be 726 μg/kg with no specifications about the type of cereals [23]; such a value is 2.1 to 3.3-folds higher than the averages reported in this study. In addition, the average of acrylamide in cereal products sold in the Hong Kong market was reported as 491 μg/kg [24] which is still 1.4- to 2.2-folds higher than the averages reported in this study. On the other hand, the average level of acrylamide among cereal products sold in the Syrian stands at 121 μg/kg; a value which is 1.8- to 2.9-folds lower than those reported in this study [25].

It is quite evident, and from the results of this study, that no direct correlation can be made between the type of cereal and acrylamide content. As acrylamide is not present in the raw material required to prepare cereal products, one can conclude that acrylamide formation might be attributed to the industrial cooking and preparation processes as well as to the asparagine levels in the original cereal grain [14]. In addition, there are no unified procedures for the preparation of cereal products and this further explains the variation in results when duplicate measurements are made on the same brand of product yet with different production dates [26]. This may suggest the lack of efficient quality control on cereal based products prior to them being sent to the market.

To further investigate the effect of cereal product type on acrylamide formation, acrylamide levels across products of different flavors including natural, honey and chocolate flavored cereals were compared. Results show that acrylamide levels in honey and chocolate flavored products were 1.5-folds higher compared to non-flavored products; a result that may be attributed to the fact that honey and chocolate flavored cereals contain larger amounts of reducing sugars. Nonetheless, this observation further validates the assumption that the industrial preparation process plays a key role in acrylamide formation.

Acrylamide levels detected in this study are also in accordance with those reported in the United Kingdom [27]; whereby cereal products with low sugar content tend to accumulate lower acrylamide levels compared to products with high sugar content. According to Hamlet, any increase in sucrose or glucose levels causes an equivalent spike in acrylamide content while a onefold increase in fructose levels causes acrylamide content to rise by as much as 20-folds. As for maltose, a onefold increase will result in swelling acrylamide levels by fivefolds. Besides the reducing sugars, it is widely and scientifically acknowledged that asparagine (Asn) plays a major role in acrylamide formation via the Maillard reaction [9, 28]. As such, cereal products with low asparagine content are highly recommended when acrylamide content is of concern to the consumer. It is also worthwhile noting that the amount of free Asn in commercial flour products [27] vary widely and consequently this would propagate to cereal products and thus further explains the variance in acrylamide content across same brand/type of product and with different production date. This natural variation means that asparagine content cannot be controlled based on grain type. However, Asn and reducing sugars do appear to vary in soft wheat and hence gristing (separation of grain from its chaff) of wheat may be worthwhile for products in which there is no added glucose or fructose in the recipe [27].

### Dietary intake of acrylamide from cereals

Evaluation of the dietary exposure to acrylamide from cereals was calculated by combining the average concentration per kg of sample and the consumption data from the survey. The dietary exposure of acrylamide from the various types of cereals was found to be 0.9 μg/kg-bw/day (corn), 1 μg/kg-bw/day (wheat), 0.7 μg/kg-bw/day (rice) and 0.7 μg/kg-bw/day (oat) and 1.2 μg/kg-bw/day (mixed) with an average of 0.7 μg/kg-bw/day. The highest intake rate was found to occur among teens (3–18 years old) consuming acrylamide from mixed cereals at a rate of 4.6 μg/kg-bw/day.

The above results lie within the range reported by the WHO which estimates the average human intake for total dietary acrylamide to vary widely between 0.3 and 2 μg/kg-bw/day [29, 30]. However, teenagers (4.6 μg/kg-bw/day) have been shown to slightly exceed the maximum risk intake of 4 μg/kg-bw/day set by the WHO in 2005 [30]. It is worthwhile noting, that the results of the investigation pertain to the dietary intake of acrylamide from cereals alone, yet the limits set by the WHO pertain to the total dietary intake of acrylamide. Taking into consideration Nasreddine’s findings in which cereals were shown to contribute 10.5% to the total acrylamide dietary intake among the Lebanese population, it becomes evident that acrylamide levels in cereal products alone sold in the Lebanese market are approximately fivefolds higher than what should be recommended based on the consumption data determined through the community survey [31, 32].

### Risk evaluation of acrylamide consumption

#### Neurotoxicity

The results of this study show that the mean exposure of the Lebanese population to acrylamide from cereal-based products is 3.5-folds (0.7 μg/kg-bw/day) higher than the US-EPA reference dose (RfD) of 0.2 μg/kg-bw/day [33]. According to JECFA, the minimum acrylamide MOEs for the mean dietary exposure is 200 and while that of the high dietary exposure is 50 [18]. Among the cereal consumers considered in this study, the acrylamide MOEs ranged from 43 to 1000; such a wide range can be attributed to the large variety in the type of cereal products (Table 2). Among all age groups, children and teenagers had MOE values below the set value of 200, for all types of cereal products, and thus are subject to chronic neurotoxic risk (Table 2). In the case of adults above 50 years of age, only consumers of oat-based cereal products have been shown to exhibit chronic neurotoxic risk. Although the average MOE values of acrylamide among the Lebanese population was found to be above 200 and thus unlikely to have adverse neurological effects for the average consumer; however, morphological changes in nerves cannot be excluded among high consumers and specifically among those consuming oat-based cereal products [18]. In order to avoid a neurotoxic risk among the entire Lebanese population as a result of cereal consumption, the maximum concentration of acrylamide should be set at 154.1 μg/kg product; a value which is 1.7 times lower than the average acrylamide concentration among the various cereal products as calculated in this study.

**Table 2:** Human exposure to acrylamide from cereal-based products

Mean acrylamide intake; μg/kg-bw/day					
Age (years)	Corn	Wheat	Rice	Oat	Mixed
Population (3–75)					
Dietary intake	0.9 ± 1.2	1 ± 1.3	0.7 ± 1.1	0.7 ± 0.9	1.2 ± 1.7
MOE_N_	222	200	286	286	167
*MOE_C_	344 (200)	310 (180)	443 (257)	443 (257)	258 (150)
Children/Teens (3–18)					
Dietary intake	2.9 ± 1.5	3.2 ± 1.8	3.1 ± 2.4	4.4 ± 6	4.6 ± 2.6
MOE_N_	69	63	65	45	43
*MOE_C_	107 (62)	97 (56)	100 (58)	70 (41)	67 (39)
Young adults (18–20)					
Dietary intake	0.4 ± 0.3	0.5 ± 0.4	0.4 ± 0.2	0.5 ± 0.4	0.7 ± 0.6
MOE_N_	500	400	500	400	286
*MOE_C_	775 (450)	620 (360)	775 (450)	620 (360)	443 (257)
Adults (21–30)					
Dietary intake	0.4 ± 0.3	0.6 ± 0.5	0.5 ± 0.3	0.7 ± 0.6	0.7 ± 0.6
MOE_N_	500	333	400	286	286
*MOE_C_	775 (450)	517 (300)	620 (360)	443 (257)	443 (257)
Adults (31–50)					
Dietary intake	0.5 ± 0.4	0.8 ± 0.5	0.4 ± 0.3	0.6 ± 0.5	0.7 ± 0.6
MOE_N_	400	250	500	333	286
*MOE_C_	620 (360)	388 (225)	775 (450)	517 (300)	443 (257)
Adults (51 +)					
Dietary intake	0.7 ± 0.6	0.2 ± 0.1	0.2 ± 0.08	1.1 ± 1	0.6 ± 0.5
MOE_N_	286	1000	1000	182	333
*MOE_C_	443 (257)	1550 (900)	1550 (900)	281 (164)	517 (300)

Dietary intake as well as the Margins of Exposure for neurotoxic risk assessment (MOE_N_) and carcinogenic risk assessment (MOE_C_) are reported. Data are expressed as μg/kg-bw/day for dietary intake. MOE_N_ values are reported for BMDL_10_ (0.2 mg/kg-bw/day). MOE_C_ values are reported for BMDL_10_ (0.31 and 0.18 mg/kg-bw/day)

#### Carcinogenicity

The results of this study reveals that the cereal-based acrylamide average exposure was 8 times higher than the NFCA estimated intake (0.08 μg/kg-bw/day) [34] and 5 times higher than the WHO set intake (0.14 μg/kg-bw/day) [35]. According to JECFA, acrylamide may pose a high carcinogenic concern if MOEs are found to occur below 310 or 180 depending on the BMDL_10_ value under consideration [18]. In terms of the entire population, as calculated from mean exposure to acrylamide from cereal products, the MOEs for carcinogenicity ranged from 67 to 1550 at BMDL_10_ (0.31 mg/kg-bw/day) and 39 to 900 at BMDL_10_ (0.18 mg/kg-bw/day). While the acrylamide MOEs for the entire population does not appear to pose a health concern, children and teens are subject to a high chronic carcinogenic risk regardless of the type of cereal product consumed with MOE values well below 100. In addition, adults (51 +) who consume oat cereals are subject to a carcinogenic risk at both BMDL_10_ values (Table 2). To avoid a carcinogenic and neurotoxic risk among the entire Lebanese population as a result of cereal consumption, the maximum concentration of acrylamide should be set at 154.1 μg/kg product. Having said that, 154.1 μg/kg product would still incur a significant risk among children in the Lebanese population due to their higher consumption rate of cereal products. To completely mitigate any risk among Lebanese children, the maximum concentration of acrylamide should be lowered by threefolds and set at 94.8 μg/kg product. Should the WHO adopt these significantly lowered proposed values, the industry will have to respond by optimizing their production process in a way to reduce and manage acrylamide levels in the production of cereal products. Alternatively, the entire Lebanese population and children among the Lebanese population may respectively choose to cut down on cereal consumption from 5.2 and 12.2 portions per week down to 3 and 1.7 portions per week should they want to avoid a health hazard as a result of acrylamide intake. Alarmingly, the EFSA (European Food Safety Authority) scientific committee has set the MOE level for carcinogenicity (and genotoxicity) to be 10,000 or lower [4]; a threshold value which is 32- and 56-folds higher than the BMDL_10_ values set by JECFA [18]. In comparison with the EFSA threshold, the results show that the entire cereal-consuming Lebanese population is subject to a high carcinogenic and genotoxic risk.

Analysis of the community survey results reveal that many of the current Lebanese population consume cereals as part of their main meals as well as breakfast. This observation further promotes for the regulation of acrylamide content in acrylamide-containing food products including cereals. Failure to do so may lead to a future generation with a high cancer rate compared to the current generation.

## Conclusion

The results of this study show that oat-based and mixed cereals contain the largest amount of acrylamide among cereal products with levels as high as 271 and 348 μg/kg respectively. While the average acrylamide intake of the entire population seems to pose no long-term health risk; children and teenagers were shown to exhibit both carcinogenic and neurotoxic risks regardless of the type of cereal product consumed. For adults above 50 years of age, only consumers of oat-based cereal products seem to exhibit carcinogenic and neurotoxic risks. To avoid a carcinogenic and neurotoxic risk among the entire Lebanese population, including children, as a result of cereal consumption, acrylamide levels should be lowered by threefolds. We also propose that food processors consider this amount of 94.8 μg/kg product as the maximum tolerable concentration for acrylamide in cereal products; and accordingly measures for quality control and assurance should be developed and adopted. Should the food processors adopt these significantly lowered proposed values, the industry will have to respond by optimizing their production process in a way to reduce and manage acrylamide levels in the production of cereal products. Alternatively, and unreasonably, the entire Lebanese population and children among the Lebanese population may choose to cut down on cereal consumption by 1.7- and 7.2-folds respectively, should they want to avoid a health hazard as a result of acrylamide intake. The call for improved and regulated industrial procedures in order to control acrylamide levels in cereal products goes hand in hand with our previous call to control acrylamide levels in caffeinated beverages sold in the Lebanese market [16].

## Data Availability

The authors declare that the manuscript contains the minimal dataset that is required to interpret, replicate, and build upon the methods and findings reported in the article. Raw data can be shared via correspondence upon reasonable request.

## Abbreviations

FDE: FoodDrinkEurope
LOQ: Limit of quantification
LOD: Limit of detection
IRB: International Review Board
bw: Body weight
MOE: Margin of exposure
NOAEL: No-observed-adverse-effect level
JECFA: Journal of the Expert Committee on Food additives
BMDL_10_: Benchmark dose for a 10% response
RfD: Reference dose
